# Avian H11 influenza virus isolated from domestic poultry in a Colombian live animal market

**DOI:** 10.1038/emi.2016.121

**Published:** 2016-12-07

**Authors:** Pedro Jiménez-Bluhm, Erik A Karlsson, Karl A Ciuoderis, Valerie Cortez, Shauna A Marvin, Christopher Hamilton-West, Stacey Schultz-Cherry, Jorge E Osorio

**Affiliations:** 1 Department of Infectious Diseases, St Jude Children's Research Hospital, Memphis, TN 38105, USA; 2 Department of Pathobiological Sciences, School of Veterinary Medicine, University of Wisconsin–Madison, Madison, WI 53706, USA; 3 Faculty of Veterinary Science, Department of Preventive Medicine, University of Chile, Santiago 8820808, Chile

**Keywords:** Colombia, H11N2, influenza, live animal market, risk assessment, surveillance

## Abstract

Live animal markets (LAMs) are an essential source of food and trade in Latin American countries; however, they can also serve as ‘hotbeds' for the emergence and potential spillover of avian influenza viruses (AIV). Despite extensive knowledge of AIV in Asian LAMs, little is known about the prevalence South American LAMs. To fill this gap in knowledge, active surveillance was carried out at the major LAM in Medellin, Colombia between February and September 2015. During this period, overall prevalence in the market was 2.67% and a North American origin H11N2 AIV most similar to a virus isolated from Chilean shorebirds asymptomatically spread through multiple bird species in the market resulting in 17.0% positivity at peak of infection. Phenotypically, the H11 viruses displayed no known molecular markers associated with increased virulence in birds or mammals, had α2,3-sialic acid binding preference, and caused minimal replication *in vitro* and little morbidity *in vivo*. However, the Colombian H11N2 virus replicated and transmitted effectively in chickens explaining the spread throughout the market. Genetic similarity to H11 viruses isolated from North and South American shorebirds suggest that the LAM occurrence may have resulted from a wild bird to domestic poultry spillover event. The ability to spread in domestic poultry as well as potential for human infection by H11 viruses highlight the need for enhanced AIV surveillance in South America in both avian species and humans.

## Introduction

Live animal markets (LAMs) represent a traditional place for congregation and commerce, particularly in developing countries. Owing to their role as a source of affordable, live or freshly slaughtered animals, LAMs act as a source for transmission of pathogens, especially viruses.^1, 2, 3^ Spread of a virus within the market is often enhanced due to high density, close contact animal housing, increasing the risk of zoonotic and anthroponotic transmission.^4^ Animals remain in the LAM for extended periods of time until sold and can consequently transform these markets into viral reservoirs. As new animals are introduced to the LAM, Infected animals easily transmit viruses to these naïve hosts, thus perpetuating and amplifying viral circulation.^5^ In addition, LAMs are often part of a larger marketplace ecosystem, potentially exposing people to zoonotic pathogens with little to no direct contact with infected animals.^1^ This is especially true with avian influenza viruses (AIV).

LAMs in many parts of the world harbor highly pathogenic as well as low pathogenic AIV, which can spread asymptomatically through poultry and are difficult to detect without routine surveillance.^6, 7, 8, 9^ Although AIV has been detected in North American and Caribbean LAMs, there is no information about South America LAMs,^10, 11^ likely due to minimal surveillance.^12^ During active surveillance at the largest LAM in Medellin, Colombia, we isolated two H11N2 viruses from asymptomatic birds. At the peak of the occurrence, 17.0% of the birds in the market tested positive. Genetically, the circulating virus was most similar to viruses isolated from North American migratory birds and to viruses isolated in 2013 from Chilean shorebirds. H11 viruses are distributed worldwide^10, 13, 14, 15^ primarily in wild ducks and shorebirds^16, 17^ but rarely are found in domestic poultry.^9, 10, 18, 19, 20^ Given this unique occurrence and the fact that H11 have been reported to cause human infections^21, 22^ we characterized the viruses *in vitro* and *in vivo*. The Colombian H11 viruses displayed no molecular markers associated with increased virulence in birds or mammals and had an α2,3-sialic acid binding preference. They replicated and transmitted effectively in chickens, explaining the spread throughout the market, but caused little morbidity in Balb/c mice. The genetic similarity to H11 viruses isolated from South American shorebirds suggests that the LAM occurrence may have resulted from a wild bird to domestic poultry spillover. These findings highlight the need for enhanced AIV surveillance in South America, especially in areas of high-risk, such as LAMs.

## Materials and methods

### Ethics statement

LAM sampling activities were performed after obtaining verbal consent from the bird owners. All animal experiments and field sampling activities were approved by the St Jude Children's Research Hospital Institutional Animal Care and Use Committee (IACUC).

### Sample site and collection

Sample collection (*n*=1160) was conducted between February and September 2015 in a LAM in Medellin, Colombia; (February, *n*=90; March, *n*=226; April, *n*=112; May, *n*=142; June, *n*=150; July, *n*= 72; August, *n*=209 and September, *n*=159). This is the only LAM that is consistently open to the public and a traditional place for people to obtain poultry and other groceries. There are five regularly established poultry sellers at the LAM and around 2500 birds are available for sale at any given time. New birds are brought to the market weekly to bi-weekly, the majority of which are supplied by backyard poultry farmers. Poultry are sold mostly alive, but can be slaughtered, de-feathered and eviscerated at the LAM upon request. Fresh environmental feces and cloacal samples were collected from individual birds and cages using single-use sterile swabs and placed in cryovials containing 1 mL Universal Transport Media, UTM (Copan Italia SPA, Brescia, Italy). Samples were kept at 4 °C for a maximum of four days then stored at −80 °C until analysis.

### Screening and virus isolation

Viral ribonucleic acid (RNA) was extracted from 50 μL sample using the Ambion MagMAX-96 AI/ND Viral RNA Isolation kit (Life Technologies Corporation, Grand Island, NY, USA) on a Kingfisher Flex Magnetic Particle Processor (Thermo Fisher Scientific, Waltham, MA, USA) as described.^23^ Influenza matrix (M) gene real-time reverse transcription PCR (RT-qPCR) was performed on a Bio-Rad CFX96 Real-Time PCR Detection System (Bio-Rad, Hercules, CA, USA) with TaqMan Fast Virus 1-Step Master Mix (Applied Biosystems, Foster City, CA, USA) and primers/probe as described.^24^ Samples with a cycle threshold value <38 were considered positive^25^ and viral isolation in embryonated chicken eggs was attempted as described.^26^ Isolates were confirmed by hemagglutination assay (HA) and RT-qPCR and viral titers determined by Reed and Munch^27^ with both 50% tissue culture infectious dose (TCID_50_) in Madin-Darby canine kidney cells (MDCK) and by 50% egg infectious dose (EID_50_). Viruses were stored at −80 °C.

### Virus sequencing

Reverse transcription of viral RNA was performed using SuperScript Vilo (Life Technologies Corporation, Grand Island, NY, USA). Amplicons were obtained using Phusion High-Fidelity DNA polymerase (New England BioLabs, Ipswich, MA, USA) with gene specific universal oligonucleotide primers as described.^28^ DNA was subsequently purified by agarose gel electrophoresis, using Zymoclean Gel DNA Recovery (Zymo Research Corporation Irvine, CA, USA). Full-length gene segments were ligated into the pCR-Blunt II-TOPO (Life Technologies Corporation) and amplified in HB101 *E. coli* strain (Zymo Research Corporation). Smaller gene fragments produced using HA1134F/HA-NS 890R primers were sequenced directly after gel purification.^29^ Sequencing was performed by Sanger sequencing at the University of Wisconsin–Madison Biotechnology Center and at the St Jude Hartwell Center using segment specific primers.^28, 29^ Host species were identified by PCR barcoding using primers designed to amplify a ~700 bp segment of the mitochondrial cytochrome-oxidase I, obtained from AIV-positive samples then sequenced as described.^30^ AIV gene segments and cytochrome-oxidase I similarities were analyzed by BLAST.^31^ The nucleotide sequences obtained in this study are available from GenBank under accession numbers KX097952 to KX097966.

### Phylogenetic analysis

Sequence assembly was performed using BioEdit version 7.2.5.^32^ Sequence alignments were generated using MUSCLE version 3.8.3^33^ and reference sequences obtained from Influenza Virus Resource at NCBI.^34^ The evolutionary history was inferred using the maximum likelihood method based on the Kimura 2-parameter model using MEGA version 6.0^35^ and the trees with the highest log likelihood are shown. Bootstrap resampling process of 500 replicates was implemented to provide statistical robustness to each node. The percentage of trees in which the associated taxa clustered together is shown next to the branches. Initial trees for the heuristic search were obtained automatically by applying Neighbor-Join and BioNJ algorithms to a matrix of pairwise distances estimated using the maximum composite likelihood approach, and then selecting the topology with superior log likelihood value. The trees were drawn to scale, with branch lengths measured in the number of substitutions per site.

### Viruses

The following viruses were used: A/duck/Memphis/546/1974 (H11N9, duck/Mem), A/ruddy turnstone/Delaware/544/2014 (H11N2, RT/DE), A/mallard/Alberta/315/2012 (H11N9, Mal/Alb), A/duck/England/1/1956 (H11N6, Duck/Eng), A/mallard/Wisconsin/11OS4115/2011 (H11N9, Mal/WI), A/mallard/Mississippi/12OS361/2012 (H11N9, Mal/MS), A/black necked stilt/Chile/2/2013 (H11N9, BNS/Chile) and A/Helmeted guineafowl/Colombia/2/2015 (H11N2, HGF/Colombia), A/duck/Ukraine/1/1963 (H3N8, Duck/Uk), A/California/04/2009 (pdmH1N1, CA/09).

### *In vitro* replication

MDCK and A549 cells (ATCC, CCL-185) were cultured in Eagle's minimum essential medium (Gibco-Invitrogen, Carlsbad, CA, USA) supplemented with 2 mM glutamine and 10% FBS (Gemini BioProducts, West Sacramento, CA, USA) and grown at 37 °C in 5% CO_2_ in a humidified atmosphere. Viral replication studies were performed as described.^23^ In brief, cells were infected at a multiplicity of infection of 0.01 for 1 h at 37 °C then washed three times to remove unbound virus and cultured in media containing 0.075% bovine serum albumin and 1 μg/mL l -1-tosylamide-2-phenylethyl chloromethyl ketone-treated trypsin. Aliquots of culture supernatants were collected at 6, 24 48 and 72 h post-infection (pi) and immediately stored at −80 °C. Viral titers were determined by TCID_50_ in MDCK cells.^27^

### Receptor-binding specificity

Receptor affinity was determined using a solid-phase direct virus binding assay as previously described.^36^ iN Brief, influenza viruses were bound to fetuin-coated plates at 4 °C overnight. Biotinylated glycans (α-2,3 or α-2,6 sialic acids, Glycotech Corporation, Gaithersburg, MD, USA) were added to influenza-coated plates at varying dilutions and incubated for 4 h and binding analyzed using horseradish peroxidase-conjugated streptavidin (Invitrogen, Carlsbad, CA, USA) followed by TMB substrate (Sigma, St. Louis, MO, USA). Absorbance (450 nm) was read on a Synergy 2 multi-mode microplate reader (BioTek Instruments, Winooski, VT, USA). K_d_ was determined by Linear Regression analysis using GraphPad Prism 5 software (La Jolla, CA, USA).

### Animal infections

Six- to eight-week-old female BALB/c mice (*n*=11, Jackson Laboratory, Bar Harbor, ME, USA) were lightly anesthetized with isoflurane and intranasally inoculated with 10^4^ TCID_50_ of virus in 25 μL PBS. Mice were monitored daily for signs of infection (body weight loss, hunched posture, ruffled coat, lethargy and dehydration) and weighed every 24 h.^37^ At day 3 and 6 pi, *n*=3 mice were euthanized and nasal washes and lungs were harvested for viral titers by TCID_50_. Chicken experiments were performed as described previously.^23^ In brief, 8-week-old specific pathogen-free chickens (*n*=5 per group) were inoculated with 10^6^ EID_50_ of virus in 0.5 mL via intraocular, intranasal and intratracheal routes, and monitored for clinical signs of infection (labored breathing, body weight loss and diarrhea) daily. One day pi, naïve chickens (*n*=5) were introduced to simulate contact transmission in a market setting. To assess virus shedding, cloacal and tracheal swabs were collected every 48 h for 12 dpi. Swabs were stored in 1 mL (cloacal) or 0.5 mL (tracheal) viral transport medium at −70 °C for virus titration by determining EID_50_ in embryonated hen eggs.

### Hemagglutination inhibition assays

Specific antisera were treated with receptor destroying enzyme (RDE; Seiken, Tokyo, Japan) and hemagglutination inhibition assays were performed according to WHO guidelines.^38^

### Statistical analysis

For *in vitro* and *in vivo* viral studies, statistical significance was determined using analysis of variance with strain and day post-infection as main effects. Logistic regression analysis was used to assess differences in AIV positivity across various bird species. A *P*-value <0.05 was considered statistically significant. Statistical analyses were performed with STATA statistical software, Version 13 (StataCorp, College Station, TX, USA), JMP statistical software (SAS, Cary, NC, USA) and GraphPad Prism software.

## Results

### Isolation of H11N2 viruses from Colombian live animal market

Given the dearth of knowledge about AIV prevalence in South American LAMs, we initiated active surveillance in a traditional LAM in Medellin, Colombia from February to September 2015. Several bird species were available for testing (Table 1), with domestic ducks, chicken and turkeys being the most frequently sampled species. AIV was first detected in March when 3/226 birds tested positive (1.3%) by RT-qPCR, peaked in April with 19/112 positive birds (17.0%), subsiding in subsequent months, indicating a self-contained occurrence. No positive samples were obtained in subsequent sampling efforts after September 2015. Unfortunately, due to accessibility issues, no serum samples were able to be collected to corroborate swabs. Based on sampling data, guinea fowl and turkeys were more likely to be AIV-positive as compared with chickens (guinea fowl: OR=10.65, 95% CI: 2.82–40.31, *P*<0.001; turkey: OR=4.47, 95% CI: 1.10–18.15, *P*=0.036). No significant differences were observed with ducks, geese or quail. No increase in morbidity or mortality was reported to us by bird owners during the sample period, and we did not notice any clinical signs consistent with influenza infection, like ruffled feathers, diarrhea, decreased activity or respiratory distress. Two H11N2 viruses, A/Helmeted guineafowl/Colombia/1/2015 and A/Helmeted guineafowl/Colombia/2/2015 (HGF/Colombia), were isolated from different birds in March 2015. In addition, partial sequences of four additional H11N2 viruses were obtained from geese and guineafowls sampled in April 2015. Sequencing and viral isolation was attempted on all samples below cycle threshold value of 35^14^ with no further success either isolating viruses or obtaining further sequence information.

### Phylogenetic and antigenic characterization

**Figure 1:**
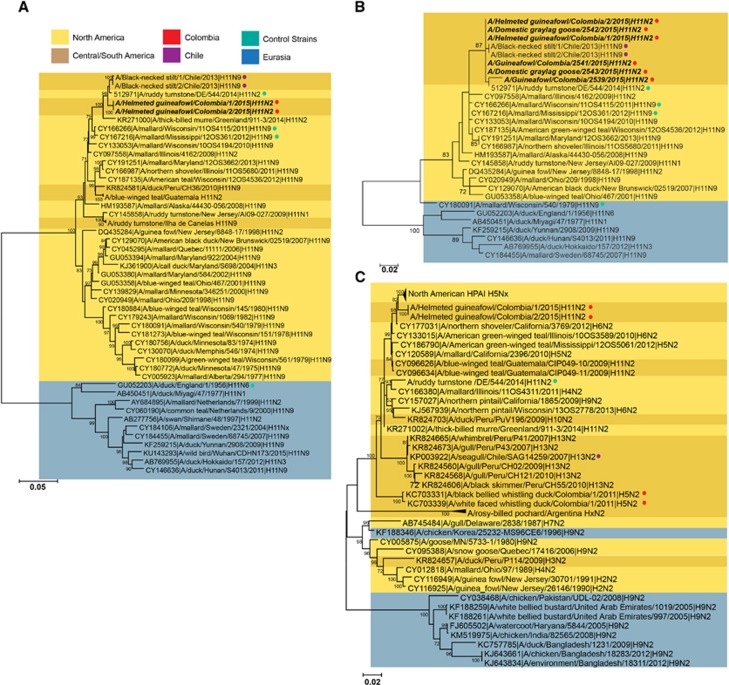
Phylogenetic trees of the hemagglutination assay (HA) composed of complete (**A**) and partial (**B**) sequences, as well as the neuraminidase (NA) (**C**) genes isolated from guineafowls at the live animal market (LAM) in Medellin, Colombia. Trees were generated using maximum likelihood method in MEGA software. Bootstrap values (*n*=500) >70 indicated. Scale bars represent substitution per sites. Strains isolated in this study are indicated in black italics. Eurasian strains, blue; North American strains, yellow; Central and South American strains, dark yellow; Colombian H11N2 sequences, red dot; Chilean H11N9 sequences, purple dot; other control strains, turquoise dot.

Full-genome sequencing was performed to determine the genetic origin of the Colombian H11N2 viruses. The Colombian H11N2 viruses were most closely related to each other and formed significantly distinct clusters from other analyzed sequences. All gene segments clustered with North American lineage AIVs, rather than with viruses belonging to South American or Eurasian lineages (Figure 1, Supplementary Figure S1) with the internal genes being 98–99% similar to the nearest North America AIVs (Table 2).

**Figure 2:**
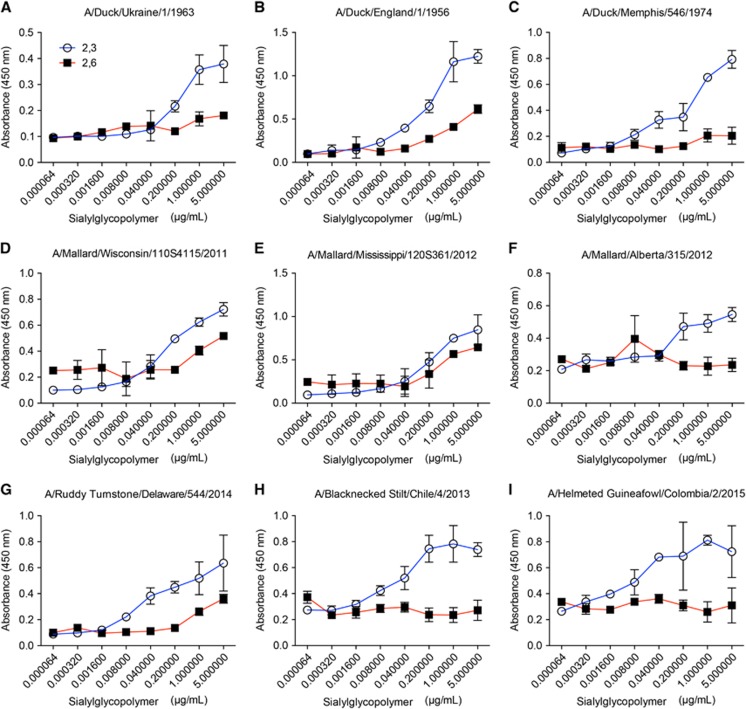
Characterization of the receptor-binding properties of isolated H11 viruses. The viruses were tested for their ability to bind to α2,3 and α2,6 sialyglycopolymers. Classical reference strains (**A**–**C**), contemporary North American strains (**D**–**G**) as well as South American strains (**H**–**I**) were included in analysis. The figure shows absorbency of the wells, versus concentration of the polymer. Error bars represent the SEM.

The HA sequences from the two isolates, as well as partial HA sequences (~400 bp) from four additional positive samples, were most similar to viruses isolated from shorebirds in Chile (A/black necked stilt/Chile/1/2013 and A/black necked stilt/Chile/2/2013, 98% nucleotide similarity) and in Delaware Bay (A/ruddy turnstone/544/2014 H11N2, 99% nucleotide similarity; Figures 1A and 1B) with the remainder coming from migratory birds in the Atlantic or Mississippi flyways (Figure 1). As expected, Colombian H11 viruses had a deduced amino acid sequence of PAIAT**R**/GLF at the multibasic cleavage site indicating the inability to replicate in the absence of trypsin. No molecular substitutions associated with mammalian host adaptation, like PB2 E627K or D701N^39,40^ were found. HA receptor-binding pocket residues (H3 numbering), at position 190, 225, 226 and 228, exhibited all avian-like amino acids that typically bind to α2,3-sialic acid receptors. In spite of position 137 displaying a human-like adaptation by presenting an arginine,^41^ solid-phase glycan-binding assays confirmed that H11 viruses had α-2,3 binding specificity (Figure 2).

Antigenically, ferret antisera generated to the Colombian and Chilean viruses had some cross-reactivity but neither virus was detected by the WHO H11 reference antisera, indicating that new reagents may need to be generated to account for H11 virus evolution in the Americas and to keep antigenic reference panels up-to-date (Table 3).

Like the HA, the neuraminidase (NA) genes of the Colombian viruses were more similar to each other than to any other strain and clustered in the North American clade of N2 viruses containing long NA stalks (Figure 1C). The nearest relative was A/northern shoveler/California/3769/2012 H6N2 (99% nucleotide similarity). The NA and the PB1 gene segments also clustered with the highly pathogenic avian influenza (HPAI) H5N2 viruses circulating in the US in 2014–2015 (Figure 1C, Supplementary Figure S1E). No changes associated with antiviral resistance were found.^42, 43^

### Colombian H11N2 transmits in chickens

Given that H11 infection in poultry is uncommon,^10, 18, 19^ we evaluated the pathogenicity and transmissibility of a panel of related H11 viruses in chickens. The Colombian H11N2 viruses are virtually identical; thus, only A/Helmeted guineafowl/Colombia/2/2015 (HGF/Colombia) was used. In brief, groups of 8-week-old, specific-pathogen-free chickens (*n*=5/virus) were inoculated by natural route with Duck/Mem, RT/DE, BNS/Chile and HGF/Colombia and monitored for clinical signs of infection. After 24 h, naïve birds (*n*=5) were housed with infected animals to monitor transmission. Cloacal and oropharyngeal swabs were collected for 12 days pi to monitor viral shed. Although none of the chickens exhibited clinical signs of disease, 100% of those inoculated with HGF/Colombia had cloacal shedding by day 2 pi with viral titers ranging from 10^4.5^ to 10^6.5^ EID_50_/mL at day 4 pi (Table 4). Chickens inoculated with the other H11 viruses also shed virus at similar titers at the peak of infection but cleared virus by day 10 pi while the HGF/Colombia virus did not clear until day 12. Similarly, HGF/Colombia infected birds exhibited oropharyngeal shedding with viral titers peaking at 10^3^–10^4.5^ EID_50_/mL by day 2 pi with clearance by day 8 pi. Intriguingly, only the Colombian virus transmitted to 60% of the contact animals by day 4 pi (Table 4). This transmission also explains the viral spread throughout the market.

### Replication of the Colombian H11N2 viruses *in vitro* and *in vivo*

**Figure 3:**
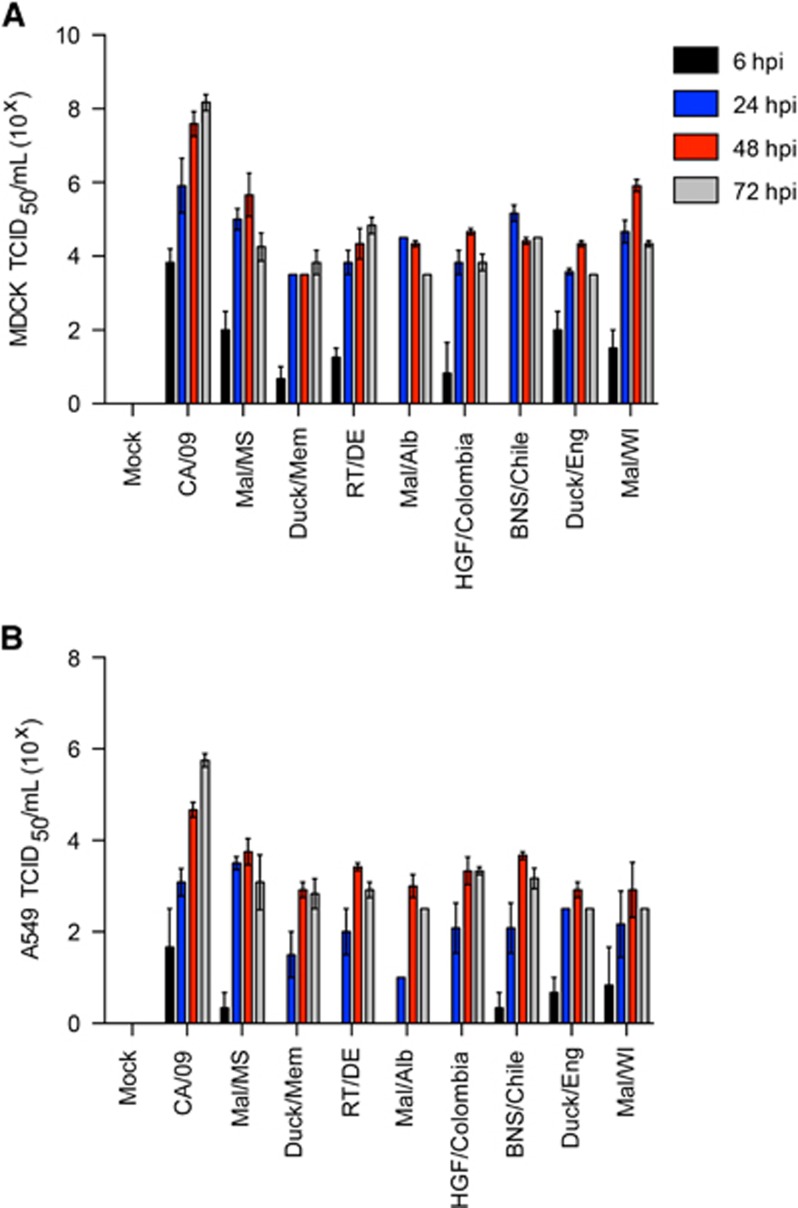
Replication of H11N2 and H11N9 viruses *in vitro*. (**A**) Madin-Darby canine kidney (MDCK) and (**B**) A549 cells were infected at a multiplicity of infection of 0.01 and supernatants were titrated as 50% tissue culture infectious dose (TDIC_50_) at 6, 24, 48 and 72 h pi. Error bars represent the SEM.

Given previous reports of human infection with H11 viruses,^21, 22^ we quantitated replication in mammalian cell lines and mice as compared with A/California/04/2009 (CA/09; pdmH1N1) virus. H11 viruses exhibited decreased replication in both MDCK (Figure 3A) and A549 (Figure 3B) cell lines as compared with CA/09 (pdmH1N1), typically being 2–3 logs lower at 48–72 h pi.

**Figure 4:**
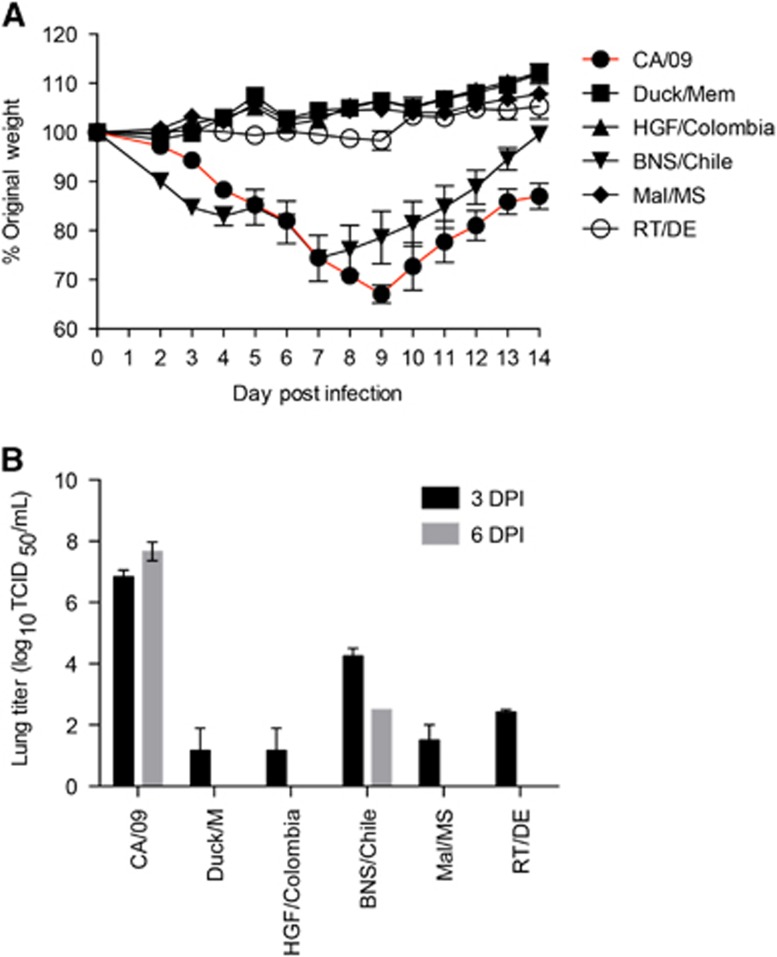
Pathogenicity of H11 viruses *in vivo*. Six to eight-week-old female Balb/c mice (*n*=11) where intranasally infected with 10^4^ 50% tissue culture infectious dose (TCID_50_) of challenge and control viruses. (**A**) Weight loss was monitored for 14 dpi and (**B**) 3 and 6 dpi, lungs were collected from three mice per virus strain and homogenates were tittered as TCID_50_. Error bars represent the SEM.

In mice, the majority of H11 viruses caused little to no weight loss and minimal viral titers were detected in the lungs at day 3 pi (Figures 4A and 4B). Interestingly, the Chilean H11N9 isolate (BNS/Chile) replicated efficiently in the lungs and induced a sharp decline in weight loss reaching 30% by 7 dpi (Figure 4A). In summary, the Colombian H11N2 viruses replicated poorly in mammalian cells and mice, suggesting minimal threat to mammals.

## Discussion

Although the H11 subtype has been found globally in wild birds,^13, 16, 44^ few studies have identified H11 viruses from domestic poultry.^10, 18, 19^ During active surveillance in a LAM in Colombia, we isolated H11 viruses from two separate birds. Phylogenic and sequence analysis of all gene segments showed their similarity to wild bird viruses of North American origin, similar to other H11 viruses obtained in Central and South America to date.^15, 45, 46^ However, unlike other poultry adapted AIVs, the N2 protein displayed a full-length stalk region.^47, 48^ The conservation of the stalk sequence implies that at the time of sampling there was still no significant adaptation of the H11N2 in *Galliformes* in spite of its transmission amongst domestic poultry at the LAM. However, future sampling efforts may pick up adaptations if these viruses continue to circulate in the LAM. Lack of significant adaptation suggests that this outbreak occurred from a recent introduction by wild birds into poultry.^49^ Interestingly, despite having the majority of characteristics of a wild bird virus, HGF/Colombia, efficiently replicated in poultry and was able to transmit to naïve contact animals, indicating a potential risk for poultry in the region.

Epidemiologically, it is extremely difficult to determine the origin of the LAM H11 viruses for several reasons. First, birds at LAM are received from commercial and backyard poultry farms located throughout Medellin and the surrounding cities, but can occasionally be imported from other provinces throughout the country. Furthermore, birds are housed in close contact and sick animals are not separated from healthy ones. The combination of multiple sources of birds, constant influx of animals and close contact make it very hard to trace the origin of the infection. One possible explanation is that the virus was introduced at the LAM *in situ*. Doves and passerines feeding on leftover grains are commonplace throughout the market and could act as potential carriers of AIV.^50^ Alternatively, many of the birds for sale at the LAM are raised in backyard flocks that are often exposed to wild birds,^51^ and could have therefore carried the virus to the LAM. As this is the first study to observe AIV in a South American LAM, future studies aimed at clarifying transmission dynamics within the markets are needed. Importantly, they should involve sampling feral birds at the LAM as well as the screening of poultry on their arrival to the market. Environmental screening of cages, floors and equipment as well as abiotic factors, like temperature and humidity, could also provide important information as to whether these contribute to the spread and maintenance of AIV in South American LAM. Our results also indicate that guineafowls and turkeys had higher odds to be infected by AIV at the LAM compared with chickens and could therefore act as sentinel species in further studies.

Serological evidence of H11 infection in humans comes from several different sources, including North American duck hunters and wildlife professionals^22^ as well as Lebanese poultry growers.^21^ These findings indicate that H11 viruses may have zoonotic potential; however, our studies suggest that the H11 viruses used in our studies have minimal risk to mammals. The majority grew inefficiently in mammalian cell lines and replicated poorly in the murine respiratory tract. With the exception of the Chilean H11 virus, little to no morbidity and absolutely no mortality was observed suggesting minimal risk. Interestingly, the Chilean and Colombian H11 isolates show different characteristics in terms of avian transmissibility as well as mammalian replication and pathogenesis. However, these viruses have very few genetic differences aside from minor changes in the polymerase and different NA subtypes. Therefore, these viruses warrant further exploration to better understand the mechanisms of H11 AIV infection in birds and mammals.

Although the distribution and characteristics of AIV in North America, East Asia and Europe have been extensively studied, the prevalence and subtype diversity of AIV in South America remains understudied in spite of recent enhanced surveillance efforts.^12, 15, 23, 52, 53, 54, 55, 56, 57^ Unfortunately, this lack of knowledge continues to confound understanding the ecology of AIV in South America and its potential public health risk. Altogether, this study is significant because it suggests that the Colombian H11N2 virus has the potential to establish itself in the poultry population. However, these viruses may not need immediate intervention due to the lack of continued viral detection after the LAM outbreak and reduced risk for mammalian infection. Overall, the paucity of data on AIV in LAMs in Colombia underscores how little is known about AIV ecology in South America, indicating the need for continued and increased active surveillance as well as production and validation of diagnostic reagents fort his understudied continent.

## Figures and Tables

**Table 1:** Prevalence of influenza viruses by species as determined by RT-qPCR

	**# Screened**	**# Positive**	**Percent positive (%)**	**95% CI**
**Order Anseriformes**	419	10	2.3	0.86–3.74
Domestic goose (*Anser anser domesticus*)	108	2	1.9	0–4.47
Domestic duck (*Anas platyrhynchos domesticus*)	311	8	2.6	0.83–4.37

**Order Galliformes**	624	21	3.4	1.98–4.82
Common quail (*Coturnix coturnix*)	5	0	0	–
Indian peafowl (*Pavo cristatus*)	11	0	0	–
Common pheasant (*Phasianus colchicus*)	22	0	0	–
Helmeted guineafowl (*Numida meleagris*)	87	9	10.3	3.91–16.69
Japanese quail (*Coturnix japonica*)	89	3	3.4	0–7.17
Turkey (*Meleagris gallopavo*)	130	6	4.6	1–8.2
Domestic chicken (*Gallus gallus domesticus*)	280	3	1.1	0–2.32

**Order Columbiformes**	15	0	0	–
Rock dove (*Columba livia*)	15	0	0	–

**Unknown (environmental)**	102	0	0	–
**Total**	1160	31	2.6	1.68–3.52

**Table 2:** Internal genes most closely related to the Colombian H11N2 viruses as established by BLAST

**Gene segment**	**Closest related virus**
*PB2*	A/white-winged scoter/ Wisconsin/10OS3922/2010 (H14N8) 99%
*PB1*	A/mallard/Maryland/14OS1447/2014 (H3N9) 99%
*PA*	A/mallard/Ohio/11OS2229/2011 (H5N2) 99%
*NP*	A/mallard/Alberta/243/2006 (H7N3) 98%
*M*	A/blue-winged teal/Texas/AI12–3566/2012 (H4N6) 99%
*NS*	A/blue-winged teal/Iowa/13OS2349/2013 (H4N8) 99%

Nucleotide identity indicated in percentage.

**Table 3:** Hemagglutination inhibition results with H11 antisera

**Viral strain**	**Subtype**	**Anti-BNS/Chile**	**Anti-HGF/Colombia**	**Anti-duck/Shan**	**Anti-duck/Eng**
A/duck/Memphis/546/1974	H11N9	<1:20	<1:20	<1:20	<1:20
A/ruddy turnstone/Delaware/544/2014	H11N2	1:40	<1:20	<1:20	<1:20
A/mallard/Alberta/315/2012	H11N9	<1:20	<1:20	<1:20	<1:20
A/mallard/Wisconsin/11OS4115/2011	H11N9	<1:20	<1:20	<1:20	<1:20
A/mallard/Mississippi/12OS361/2012	H11N9	<1:20	<1:20	<1:20	<1:20
A/black necked stilt/Chile/2/2013	H11N9	**1:160**	1:20	<1:20	<1:20
A/Helmeted guineafowl/Colombia/2/2015	H11N2	1:40	**1:80**	<1:20	<1:20
A/duck/Shantou/1411/2000	H11N2	<1:20	<1:20	**1:320**	<1:20
A/Duck/England/56	H11N6	<1:20	<1:20	<1:20	**1:160**

Abbreviations: black necked stilt, BNS; helmeted guineafowl, HGF. Homologous titers are represented in bold.

**Table 4:** Growth and transmission of Colombian H11N2 viruses in chickens

**2 dpi**		**4 dpi**		**6 dpi**		**8 dpi**		**10 dpi**	
**Direct**	**Con**	**Direct**	**Con**	**Direct**	**Con**	**Direct**	**Con**	**Direct**	**Con**
**Duck/Mem**									
100%^a^ (2.5–4)	ND^b^	100% (2.5–3)	ND	ND	ND	ND	ND	ND	ND
100%^c^ (4–5)	ND	100% (4.5–6)	20% (2.5)	40% (3.5–4)	ND	20% (2.5)	ND	ND	ND
									
**RT/DE**									
40%(3–4)	ND	60% (2.5–3)	ND	20% (2.5)	ND	ND	ND	ND	ND
60% (3.5–4.5)	ND	60% (3.5–4.5)	20% (2.5)	40% (2.5–3.5)	ND	20% (2.5)	ND	ND	ND
									
**BNS/Chile**									
100% (2.5–4.5)	ND	60% (2.5)	ND	ND	ND	ND	ND	ND	ND
100% (5–6.5)	ND	80% (4.5–5.5)	ND	80% (2.5–4.5)	ND	20%(2.5)	ND	ND	ND
									
**HGF/Colombia**									
100% (3–4.5)	ND	100% (2.5–3.5)	ND	40% (2.5)	ND	ND	ND	ND	ND
100% (4.5–6.5)	ND	100% (4.5–6.5)	60% (2.5–4.5)	80% (3.5–5.5)	60% (2.5–4.5)	40% (2.5)	20% (2.5)	20% (2.5)	ND

Abbreviations: black necked stilt, BNS; Delaware, DE; days post-infection, dpi; 50% egg infectious dose, EID_50_; helmeted guineafowl, HGF; Memphis, Mem; not detected, ND; ruddy turnstone, RT.

a Percent of animals shedding oropharyngeal route. Parenthesis indicates the viral titers in log10 EID_50_/mL. Data are the average of five animals/group.

b Values were below the limit of detection (<1 log_10_ EID_50_/100 μL).

c Percent of animals shedding cloacal route. Parenthesis indicates the viral titers in log10 EID_50_/mL. Data are the average of five animals/group.
