# Supplementary figures and images for: Avian H11 influenza virus isolated from domestic poultry in a Colombian live animal market
Source: Emerg Microbes Infect. 2016 Dec 7;5(12):e121–. doi: 10.1038/emi.2016.121 (PMC5180366; doi:10.1038/emi.2016.121)

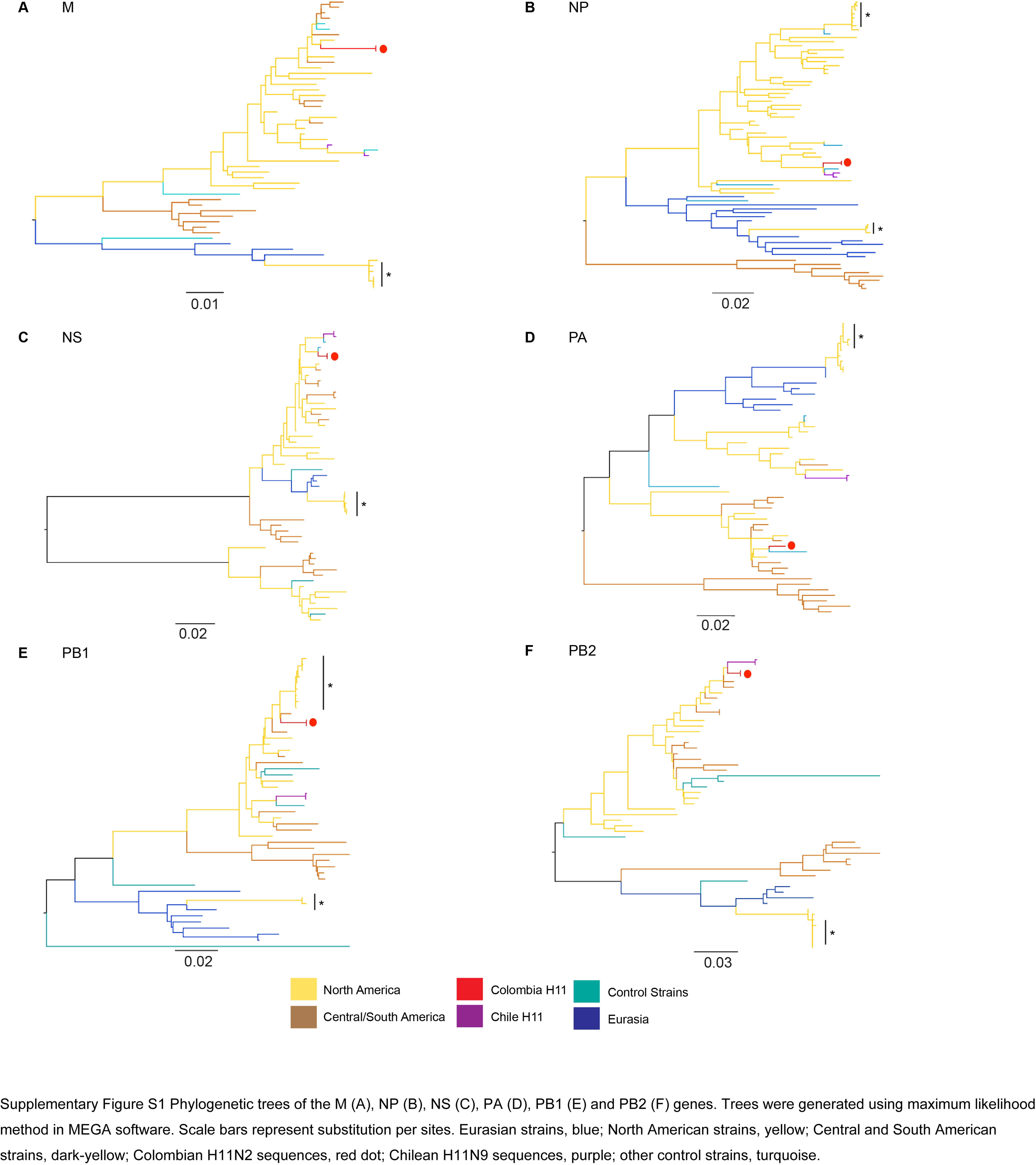

Supplement: Supplementary Figure S1 [file emi2016121x1.tif]
